# Patient-centered research: how do women tolerate nipple fluid aspiration as a potential screening tool for breast cancer?

**DOI:** 10.1186/s12885-022-09795-8

**Published:** 2022-06-27

**Authors:** Susana I. S. Patuleia, Cathy B. Moelans, Jasmijn Koopman, Julia E. C. van Steenhoven, Thijs van Dalen, Carmen C. van der Pol, Agnes Jager, Margreet G. E. M. Ausems, Paul J. van Diest, Elsken van der Wall, Karijn P. M. Suijkerbuijk

**Affiliations:** 1 Department of Pathology, University Medical Center Utrecht, Utrecht University, Utrecht, The Netherlands; 2 Department of Medical Oncology, University Medical Center Utrecht, Utrecht University, Utrecht, The Netherlands; 3 Department of Surgery, Diakonessenhuis Utrecht, Utrecht, The Netherlands; 4 Department of Surgery, Erasmus MC Cancer Institute, Rotterdam, The Netherlands; 5 Department of Surgery, Alrijne Hospital, Leiderdorp, The Netherlands; 6 Department of Medical Oncology, Erasmus MC Cancer Institute, Rotterdam, The Netherlands; 7 Division Laboratories, Pharmacy and Biomedical Genetics, Dept. of Genetics, University Medical Center Utrecht, Utrecht University, Utrecht, The Netherlands

**Keywords:** Nipple aspirate fluid, Nipple fluid aspiration, Blood, Screening, Breast cancer, Liquid biopsies, Tolerability, Discomfort

## Abstract

**Background:**

Nipple fluid aspiration (NFA) is a technique to acquire nipple aspirate fluid (NAF), which is considered a rich source of breast-specific biomarkers. Originating directly from the mammary ducts, this liquid biopsy can offer insight into the process of carcinogenesis at its earliest stage and therefore could be of added value to the current imaging-based breast cancer screening tools. With that in mind, it is necessary to know how well NFA is tolerated.

**Aim:**

To evaluate the participants’ tolerability of NFA compared to breast imaging screening methods and blood draws.

**Materials and methods:**

Three cohorts of women underwent NFA: healthy women (*n* = 190), women diagnosed with breast cancer (*n* = 137) and women at high risk of developing breast cancer (*n* = 48). A 0–10 discomfort score of NFA, mammography, breast MRI and blood draws, was filled in at the study visits, which took place once or annually.

**Results:**

The median discomfort rate of NFA was 1, which was significantly lower than the median discomfort of mammography and breast MRI (5 and 3, respectively, *p* < 0.001), but significantly higher than median discomfort for blood draws (0, *p* < 0.001). The great majority of women would undergo the procedure again (98%) and recommend it to others (97%).

**Conclusion:**

This study shows that NFA was well tolerated by healthy women, women diagnosed with breast cancer and high-risk women. This makes NFA a feasible method to pursue as a potential future breast cancer early detection tool, based on resident biomarkers.

**Trial registration:**

NL41845.041.12, NL57343.041.16 and NL11690.041.06 in trialregister.nl.

**Supplementary Information:**

The online version contains supplementary material available at 10.1186/s12885-022-09795-8.

## Introduction

Breast cancer is the most common cancer amongst women [1] and detection at an early stage is key for better treatment and survival outcomes [2–4]. Well-established imaging-based population and high-risk screening programs [5–11] have contributed to improved early breast cancer detection. Still, a significant proportion (12–47%) of the diagnosed invasive breast tumors and ductal carcinoma in situ tumors are not diagnosed at scheduled screening visits [12, 13]. Therefore, efforts focus on the development of tools beyond current imaging-based screening. In that context, liquid-based biomarkers for early detection of breast cancer are being investigated as these have the potential to be implemented as an add-on or triage tool in the early detection workup [14]. Liquid-biopsy based screening holds promise as it allows repeated sampling by non-invasive means with potential high accuracy, simple and fast interpretation at low costs [15, 16]. This could especially be of value for women with a higher chance of false negative results, such as women with dense breasts [17]. Moreover, mammography is generally perceived as uncomfortable and painful, which can lead to reluctance to comply to screening guidelines [18, 19]. Liquid biopsy-based screening could potentially be more tolerable than imaging-based screening and, as such, reduce the threshold for women to attend screening.

A specific liquid biopsy of the breast is nipple aspirate fluid (NAF), a biofluid that accumulates at small amounts in the breast ducts of non-lactating women [14]. As such, it can provide information about the breast microenvironment and its subtle changes. The possibility of synchronous acquirement of matched pairs of bilateral NAF samples makes this liquid biopsy, from a research point of view, even more interesting as it provides an intra-patient control for unilateral disease. The collection technique is called nipple fluid aspiration (NFA), a non-invasive technique that was first described by George Papanicolaou [20, 21], the developer of the Pap smear test which is widely used in early detection of cervical cancer. The NFA procedure uses a manual vacuum breast pump to obtain breast fluid from the duct openings of the nipple after oxytocin nasal spray stimulation [22, 23]. While results from ongoing studies investigating the potential role of biomarkers found in NAF await [24–26], it is vital to investigate the tolerability of NFA by the most relevant stakeholders: women.

We previously published the feasibility results of (repeated) NFA in separate cohorts of healthy women [22] and high-risk women [23, 27], including adherence to study visits and discomfort associated with the procedure. Here, we provide an update of the discomfort scores of NFA, now also compared to blood draw, in three cohorts at different stages of the breast cancer care pathway: healthy women at population risk undergoing population screening, women diagnosed with breast cancer and high-risk women undergoing intense surveillance.

## Materials and Methods

### Study cohorts, setting and ethics

Three cohorts of women were included in the present analysis: healthy women, women with breast cancer and women at high-risk of developing breast cancer. These cohorts are all part of the Dutch Nipple Aspirate Fluid project [24–26, 28]. Healthy women and women with breast cancer underwent one study visit, whereas high-risk women underwent sequential visits with a preferential regularity of 1 year in between, according to the Dutch national screening guidelines.

Inclusion of the healthy cohort [26] was started in August 2017 and closed in February 2021. Only women who were 45 years or older, did not have breast cancer and were not at increased risk for developing breast cancer according to personal and familial history were eligible. Women were recruited by word of mouth, radio announcement and flyers at general practitioner offices, the University Medical Center Utrecht (UMCU) hospital, breast cancer screening units and blood banks, amongst others. A total of 190 women were included.

In the second cohort, breast cancer patients [25] were included from January 2017 until March 2021. Patients were identified at multidisciplinary expert team meetings of four hospitals: UMC Utrecht, Alexander Monro hospital, Diakonessenhuis hospital and Alrijne hospital. Patients having untreated primary invasive breast cancer were eligible to participate. A total of 137 women were included.

Women at high risk of developing breast cancer and undergoing routine surveillance were included in the high-risk cohort [24] between May 2017 and February 2020; yearly study visits were performed until March 2021 at the UMC Utrecht. This subcohort (*n* = 48) of high-risk women was selected based on overlapping study participation period with the healthy and breast cancer cohorts and comprises consecutive inclusions of high-risk women, except for two women with a history of invasive breast cancer, who were excluded from the analyses. Participants were informed about the study at the outpatient clinics of the genetics department and familial cancer clinic in the UMC Utrecht. Inclusion criteria comprised having a cumulative lifetime risk (LTR) higher than 20% estimated at the genetics department at the time of inclusion [29] as described in Table S1.

Exclusion criteria for all cohorts comprised active breast infection, pregnancy and lactation. See File S1 for specified inclusion and exclusion criteria per cohort. Written informed consent was obtained from all participants and the ethical review boards within the participating hospitals approved the studies (NL41845.041.12, NL57343.041.16 and NL11690.041.06).

### Nipple fluid aspiration and blood collection

Study visits were carried out by trained research nurses, and included NFA, phlebotomy and questionnaires. Performing NFA requires a short practical training and can be easily learned. Participants were asked to apply an anesthetic cream onto the nipple covered with an occlusive plaster prior to the study visits (for at least one hour), to minimize discomfort due to the NFA. At the study visit, participants were seated in an upright position. The trained research nurse cleansed the breast, smeared a scrub gel to remove any occluding keratin plugs and disinfected the nipple with alcohol. This was followed by inhalation of one spray of oxytocin nasal spray in both nostrils, in a dose of 4 IU per spray, to stimulate NAF flow towards the nipple. Subsequently, a suction cup (also known as modified Sartorius cup [30]) was placed over the nipple attached to a plastic tube and a 50 cc syringe (Figure S1a). Repeated gentle plunger withdrawal of the syringe created a vacuum around the breast and led to the flow of NAF to the nipple surface. When a droplet appeared, the cup was detached and the fluid droplets were collected from the nipple surface using glass capillaries (Figure S1b). The procedure was defined as successful when droplets were visible on the nipple surface and could be collected. A total of three attempts were performed within a maximum of 20 min per breast. Vacuum suction was then repeated on the other breast. The obtained nipple fluid was air flushed with a pipet from the capillary into barcoded biobanking tubes, after which the capillary was flushed with 10 µl of RLT plus buffer (Qiagen, Venlo, The Netherlands; supplemented with 1:100 v/v beta-mercaptoethanol) to remove all remnants from the capillary wall. The volume of the sample was then estimated by comparing the NAF (including buffer) volume obtained with the volume in biobanking tubes previously prepared with several predefined volumes (10 µL, 20 µL, 30 µL, 40 µL and 50 µL). Samples were immediately stored at -80 °C. NAF characteristics like volume, color and viscosity [31], together with duration of NFA, who performed the NFA and the breast side order of NFA procedure were registered.

Blood was collected by phlebotomy in the median cubital vein. After collection, serum (in BD Vacutainer SST II Advance tubes) and plasma (in BD Vacutainer K2E (EDTA) tubes) were processed within 1 h by centrifugation at 1500 × g for 20 min. Aliquots of serum (*n* = 10) and plasma (*n* = 6) were immediately biobanked at -80ºC. Blood collection was initially not part of the high-risk cohort study protocol, and hence, not included in the discomfort assessment of our previous studies [22, 23, 27].

### Questionnaire

After blood and bilateral NAF collection, participants completed a discomfort questionnaire that we adapted from a questionnaire first described by Klein et al.[22, 23, 27, 32]. Discomfort regarding NFA, blood collection, breast surveillance techniques (mammography and breast MRI), breast physical exam and breastfeeding was scored on a scale from zero (no discomfort) to ten (worst discomfort imaginable); see File S2. Breastfeeding and breast physical exam were included as a means of comparison; the first because it is comparable to the NFA procedure and as such might cause a similar discomfort, the latter because the breast exposure by itself can cause discomfort. It was chosen to evaluate discomfort rather than pain to facilitate comparison with our previous studies, but also due to its applicability for all these techniques and circumstances as the word ‘discomfort’ engulfs more (implicit) aspects such as e.g. embarrassment and duration of a technique. For instance, discomfort associated to a breast physical exam is possibly associated to breast exposure and physical contact; pain would not be an applicable measure to assess this variable. Additional questions comprised whether participants would undergo the NFA procedure again and recommend it to others. In 2019, the question whether participants would accept NFA as a screening tool was added to the questionnaires.

### Statistical analyses

Statistical analyses were performed using IBM SPSS Statistics for Windows version 25.0.0.2 (IBM Corp., Orchard Road Armonk, New York, US) and GraphPad Prism for Windows version 8.0 (GraphPad Software, La Jolla, California, US). A two-tailed p-value < 0.05 was considered statistically significant. Data are presented as median with interquartile range (IQR) or mean with standard deviation (SD) when appropriate for continuous data, and counts with percentages for categorical data. Normality of data distribution was evaluated by Kolmogorov–Smirnov test.

The chi-square test was used to compare the binary variables breastfeeding, mammography, breast MRI, blood collection, breast MRI, breast physical exam (yes vs. no) between cohorts. The Mann Whitney test was used to compare age between cohorts. The Mann–Whitney test was used to compare median discomfort of NFA with the median discomforts of breastfeeding, breast physical exam, mammography, breast MRI and blood collection. Violin plots were made in GraphPad to display experienced discomfort distribution and medians. In the high-risk cohort, discomfort scores of the first study visit were included in the analyses. The Hodges-Lehmann Estimate (HLE) [33] was used to compare the median discomfort of NFA with the discomfort of mammography, breast MRI, blood collection, breastfeeding and breast physical exam and significance was calculated with Wilcoxon signed rank test. Discomfort scores between study visits in the high-risk participants were compared by Wilcoxon signed ranks tests. Of note, the variable now used to report discomfort of NFA was defined as the discomfort experienced by the vacuum created by the modified breast pump, while in our previous studies we reported mean discomfort of several aspects of the study visit, including discomfort of waiting, filling in a questionnaire, NFA and the nose spray [27].

The Spearman’s test was used to investigate correlation between discomfort scores with age, parity, breastfeeding, duration of breastfeeding, history of spontaneous nipple fluid discharge, breast size, use of contraception, age at menarche, menopausal status, NAF sample volume, duration of the NFA and successful NFA (at least one droplet). A significance value below < 0.05 was deemed necessary to indicate a correlation and a minimum of 0.7 was considered a lower bound value to indicate a strong correlation between variables. For logistic regression analyses, the enter method was used and NFA discomfort was dichotomized into ‘no discomfort’ (scores 0–3) and ‘discomfort’ (scores 4–10). A significance value below < 0.05 was deemed necessary for a variable to indicate a relevant effect on NFA discomfort.

## Results

### Cohort characteristics

A total of 375 women of three cohorts were included in the study (Table 1). Median age was 54 years old (IQR 47–62). A total of 250 women had experienced a breast physical exam, 254 had breastfed, 319 had undergone a mammography, 114 a breast MRI and 358 underwent a blood draw in the context of the study visit (Table 1).

**Table 1:** Cohort characteristics

Cohort	All	Healthy volunteers cohort	Breast cancer cohort	High-risk cohort	*P* value	*P* value	*P* value
					HC vs. BC	BC vs. HR	HC vs. HR
*n*	375	190	137	48			
Age, median (Q25-Q75)	54 (47–62)	54 (49–60)	56 (49.5–67.5)	36 (27–48)	0.093	< 0.001	< 0.001
Mammography, *n*	319	154 (81%)	137(100%)	31 (62%)	< 0.001	< 0.001	< 0.002
Breast MRI, *n*	114	3 (2%)	76 (56%)	36 (72%)	< 0.001	0.034	< 0.001
Blood collection, *n*	358	184 (96.8%)	130 (94.9%)	44 (91.7%)	0.950	0.082	0.064
Breast physical examination, *n*	250	92 (48%)	112 (82%)	43 (86%)	< 0.001	0.563	< 0.001
Ever breastfed, *n*	254	139 (73%)	91 (66%)	27 (56%)	0.245	< 0.001	< 0.001

In the high-risk cohort, only data from the first visit was considered for this baseline table. Abbreviations: *Q25-Q75* Inter-quartile range between quartile 25 and quartile 75, *MRI* Magnetic resonance imaging, *HC* Healthy volunteers cohort, *BC* Breast cancer cohort, *HR* High-risk cohort

Women in the healthy volunteer and breast cancer cohorts were significantly older than women in the high-risk cohort (both *p* < 0.001; Table 1). In the three cohorts, the majority of women had undergone a mammography (Table 1). Only 2% of the women in the healthy volunteer cohort had experienced a breast MRI, whereas 56% of the breast cancer cohort and 72% of the high-risk cohorts had undergone a breast MRI. Blood was obtained in 95.5% of all participants.

In the three cohorts, the majority of women were parous (83%, 82% and 62% of women in the healthy, breast cancer and high-risk cohorts, respectively) (Table S2). From the parous women, 89%, 81% and 87% had breastfed in the healthy, breast cancer and high-risk cohorts, respectively. Most of the women in the breast cancer cohort (82%) and high-risk cohort (86%) had experienced a breast physical exam, in contrast to the healthy volunteers, of whom 48% had undergone a breast physical exam.

Within the high-risk cohort, participants underwent a NFA between 1 to 4 times (median study visits = 1, IQR = 1–2) with a median time of one year in between visits (median 384 days; IQR 363–499).

#### Discomfort of nipple fluid aspiration compared to mammography, breast MRI, blood collection, breast physical exam and breastfeeding

**Fig. 1:**
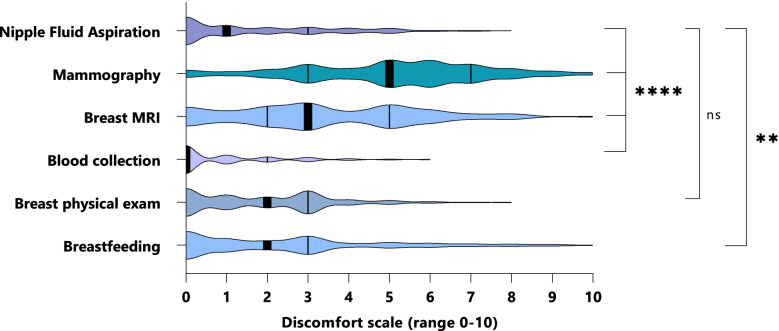
Discomfort of nipple fluid aspiration (NFA) compared to mammography, breast MRI, blood collection, breast physical exam and breastfeeding, in all cohorts. The thickened line in each violin plot represents the median. Abbreviations and symbols: MRI: magnetic resonance imaging; ns: not significant; **** *p*-value < 0.0001; ** *p*-value < 0.01 statistically significant. Significances were calculated with the Mann Whitney U test

Overall, NFA discomfort scored a median of 1 on a scale from 0–10. NFA discomfort scores were significantly lower compared to breastfeeding (median = 2; *p* = 0.001), breast MRI (median = 3, *p* < 0.001) and mammography (median = 5; *p* < 0.001); NFA scores were nevertheless significantly higher compared to discomfort of blood collection (median = 0; *p* < 0.001; see Fig. 1 and Table S3). There was no significant difference between the discomfort experienced by NFA and a breast physical exam (median = 2; *p* = 0.057). These results were confirmed with a Hodges-Lehmann Estimate analysis (HLE, Table S4). To put these data in perspective, 72% and 41% of the women gave a discomfort score of 4 or higher to mammography and breast MRI, respectively, whereas this was only the case for 21% of the women regarding NFA (Table S5).

NFA was assigned a significantly lower discomfort score in the healthy volunteers cohort (median = 1) and the breast cancer cohort (median = 1), compared to the median NFA score of 3 in the high-risk cohort (*p* = 0.005) (Table S3). To understand whether this could be related to other variables, correlation analyses were performed, but there were no clinically relevant correlations between baseline factors and NFA discomfort (all rho/coefficients < 0.3, Table S6). As the high-risk cohort had a significant younger age compared to the other cohorts, discomfort scores between cohorts were then compared, correcting for age, with a hierarchical logistic regression analysis. With this correction, there was no significant difference between cohorts regarding NFA discomfort scores (Table S7). A stratification analysis by age groups further supported this as it showed that NFA discomfort medians significantly diminish with older age (*p *< 0.001, Table S8a). As such, the higher discomfort scores of NFA in high-risk women could at least partly be explained by their younger age. The higher tolerability of older women to undergo NFA is also strengthened by the fact that NFA duration was significantly longer for postmenopausal women compared to premenopausal women (Table S8b).

Within cohorts, discomfort of NFA was consistently lower than in the case of mammography (*p* < 0.0001 in the three cohorts) and breast MRI (*p *< 0.008 and *p* < 0.0001 in the high-risk and breast cancer cohort, respectively). NFA discomfort was consistently higher than blood collection (*p* < 0.002) within cohorts. Discomfort regarding breastfeeding and breast physical exam was not significantly different compared to the discomfort rates of NFA within the breast cancer cohort and the high-risk cohort (Table S3).

Repeated NFAs in high-risk participants did not significantly affect the NFA discomfort score (Table S9). Almost all participating women would opt for repeated NFA (98%, *n* = 311/318) and would recommend NFA to others (97%, *n *= 308/317). Additionally, we asked whether women would endorse NFA in the context of screening, and most responses were positive (95%, *n* = 36/38), if proven effective (Table S10). In the high-risk group, 13 women dropped out (27%) due to breast cancer diagnosis, bilateral prophylactic mastectomy or because they found NFA unpleasant or were disappointed by the volume obtained, amongst other reasons (Table S11). Reported adverse events in all cohorts were rare (2% of all the NFA procedures) and self-limiting. These comprised fainting after blood collection or NFA, hematoma, headache and abdominal cramps (Table S12).

## Discussion

Early involvement of women in research related to the development of new breast cancer screening tools is essential. One upcoming line of investigation involves the integration of liquid biopsies as a minimally invasive, safe and sensitive additive early detection tool [16, 17, 34]. Even though there is a great number of articles on the subject of biomarkers in liquid biopsies for early detection of breast cancer, there are almost no studies reporting the women’s perspectives about the different aspects that come with the scenario of introducing liquid biopsies in screening. Here, we assessed how women from three different cohorts (healthy women undergoing or about to undergo population screening, women diagnosed with breast cancer and women at high risk of breast cancer undergoing intensive surveillance) experienced liquid biopsy acquisition (NAF and blood) in comparison to breast imaging.

Discomfort experienced during the NFA technique was significantly lower (median 1) than the discomfort experienced from the conventional screening/diagnostic tools mammography and breast MRI. The discomfort scores presented here are comparable to our previous reports in other cohorts of healthy women [22] and high-risk women [23, 27] (mean discomfort of 1.3, 0.6 and 0.71, respectively). Another study from Klein et al*.* [32] reported a higher score (median = 2; range = 1–7) for the discomfort associated to NFA compared to the cohorts in the present study, when taken together. Those higher scores were acquired from a cohort with fewer and younger women (25 healthy women with a mean age of 38 years old) compared to our three cohorts altogether. Since we show that younger age is associated with higher NFA discomfort, this might explain the observed difference in discomfort. Within all cohorts, NFA had consistently significantly lower discomfort scores than mammography and breast MRI which are now widely used screening/diagnostic techniques. Given the overall low discomfort scores, NFA, could be widely applicable for the general female population.

As a means of comparison between liquid biopsies, discomfort of blood collection, the most regular applied source of liquid biopsy, was taken along in our analyses. Blood collection is a fast, widely performed and accepted procedure that only requires exposure of the arm. As such, its discomfort scored unsurprisingly the lowest. Still, the advantages of NAF as a valuable information source for biomarkers about alterations in intraductal health, together with information about breast side and intra-patient control, have the potential to surpass the advantages of blood-based biomarkers. Such studies are under way [24–26].

The high (intended) willingness of the women in our cohorts to participate again is consistent with our previous studies [22, 23, 27]. Interestingly, a recently published questionnaire-based study reported data of 3178 women about awareness, information and preferences about breast cancer screening, including questions regarding knowledge about nipple aspirate fluid [35]. When asked about their willingness to produce NAF samples themselves at home, over 70% of the respondents would be willing to do so. The great majority of the respondents in this study were under the age of 50 and had never underwent a NFA themselves and 88% had never heard or were not aware of nipple fluid aspiration. One other study showed that there is a great willingness of women to provide liquid biopsies (blood and saliva) for biobanking at the time of breast cancer screening [36]. Altogether, these studies show that the use of nipple aspirate samples, including the NFA approach, would be very well accepted by women and is complementary to our data from women who underwent NFA.

The presented results might be influenced by self-selection bias. That is, by participating in the study, women are already willing to collect NAF and blood. Similarly, women who previously experienced a high discomfort during mammography and/or breast MRI, could be more motivated to participate in our study. Still, our discomfort scores for mammography are in line with the reported means for pain in other studies, which vary between 4 and 7 (scale 0–10) [37–39]. While discomfort associated to mammography is mostly associated to pain provoked by the plates that compress the breasts, discomfort associated to breast MRI is a result of intravenous contrast injection and lying on an MRI table while being subject to intermittent loud noises for about one hour [40–44]. And while these breast imaging disadvantages have been reported by women who have undergone these imaging techniques, it could be a reason for women to not comply to screening. As such, low discomfort associated to liquid biopsy acquisition could possibly represent an opportunity to offer a surveillance alternative at a low threshold for women who experience the imaging techniques as a burden.

## Conclusions

In summary, NFA and blood collection are simple, minimally invasive, repeatable methods that are very well accepted and endured by women. The great majority of participating women would undergo NFA again in the context of research and screening. These data indicate that a liquid biopsy biomarker-based (repeated) surveillance testing tool could be well received by women.

## Supplementary Information

**Additional file 1. Figure S1a.** Picture of syringe attached to the plastic tube on one end and to the plastic cup on the other end.

**Additional file 2. Figure S1b.** Picture of a glass capillary used to collect nipple fluid droplets. 

**Additional file 3. File S1.** Eligibility criteria per cohort.

**Additional file 4. Supplementary File 2. **Discomfort questionnaire 

**Additional file 5. Supplementary Tables 1-12**. (**S1**) Inclusion reason in the high-risk cohort, (**S2**) Cohort characteristics in breast cancer, healthy volunteers and high-risk cohorts, (**S3**) Discomfort of nipple fluid aspiration (NFA) compared to breast physical exam, breastfeeding, mammography, breast MRI and blood collection in the breast cancer cohort, healthy volunteers cohort and high-risk cohort, (**S4**) Hodges-Lehmann Estimate (HLE) of discomfort scores for nipple fluid aspiration (NFA), mammography, breast MRI, breast physical exam, breastfeeding and blood collection, (**S5**) Percentage of women of all cohorts who reported a discomfort score of 5 or above for NFA, mammography, breast MRI, breast physical exam, breastfeeding and blood collection, (**S6**) Correlation tests comparing NFA discomfort with other variables and significance values from the linear regression analyses, (**S7**) Logistic regression showing that, when corrected for age, NFA discomfort scores are not significantly different between cohorts, (**S8a**) Nipple fluid aspiration discomfort scores by age in all cohorts, (**S8b**) Median duration of nipple fluid aspiration by menopausal status in all cohorts, (**S9**) Association between discomfort NFA and repeated NFA in the high-risk cohort, (**S10**) Response of women in all cohorts and specifically in the breast cancer, healthy volunteers and high-risk cohorts,(**S11**) Drop-out reasons in high-risk cohort, (**S12**) Adverse events in all cohorts and specifically in the breast cancer, healthy volunteers and high-risk cohorts.

## Data Availability

The authors confirm that the data supporting the findings of this study are available within the article and its supplementary materials.
