# Supplementary figures and images for: Patient-centered research: how do women tolerate nipple fluid aspiration as a potential screening tool for breast cancer?
Source: BMC Cancer. 2022 Jun 27;22:705. doi: 10.1186/s12885-022-09795-8 (PMC9235076; doi:10.1186/s12885-022-09795-8)

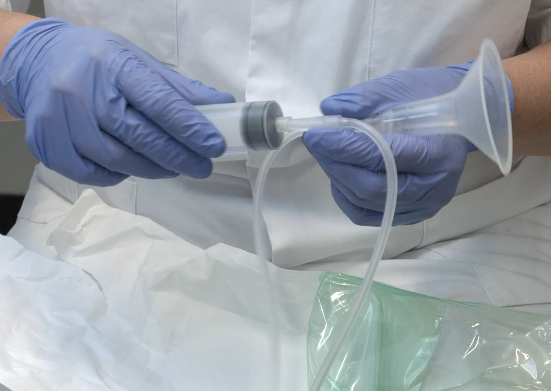

Supplement: Supplementary file 1 — Additional file 1. Figure S1a. Picture of syringe attached to the plastic tube on one end and to the plastic cup on the other end. [file 12885_2022_9795_MOESM1_ESM.png]

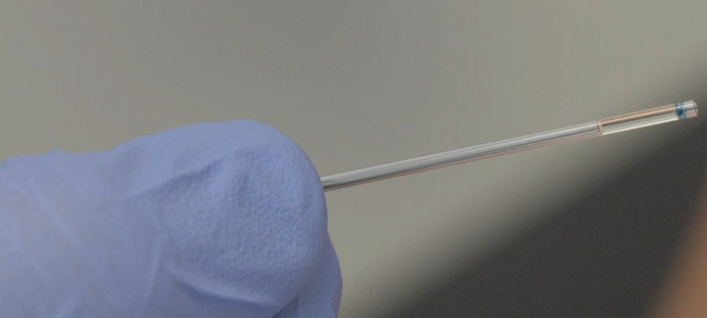

Supplement: Supplementary file 2 — Additional file 2. Figure S1b. Picture of a glass capillary used to collect nipple fluid droplets. [file 12885_2022_9795_MOESM2_ESM.png]
