# Supplementary material for: Patient-centered research: how do women tolerate nipple fluid aspiration as a potential screening tool for breast cancer?
Source: BMC Cancer. 2022 Jun 27;22:705. doi: 10.1186/s12885-022-09795-8 (PMC9235076; doi:10.1186/s12885-022-09795-8)
Supplement: Supplementary file 3 — Additional file 3. File S1. Eligibility criteria per cohort. [file 12885_2022_9795_MOESM3_ESM.docx]

## Supplementary File S1 – Eligibility criteria for the three cohorts

## Eligibility criteria for breast cancer cohort

| **Inclusion Criteria** | **Exclusion Criteria** |
| --- | --- |
| Patients must meet ***all*** of the following criteria ***to be eligible*** for enrollment as study participants: | Patients who meet ***any*** of these criteria are **not *eligible*** for enrollment as study participants: |
| - Female, age ≥18 years - Proven invasive breast carcinoma | - Bilateral breast reduction with nipple graft - Bilateral ablative breast surgery - Pregnancy or lactation - Active breast infection - Disseminated breast cancer |

**Eligibility criteria for healthy volunteers cohort**

| **Inclusion Criteria** | **Exclusion Criteria** |
| --- | --- |
| Patients must meet ***all*** of the following criteria ***to be eligible*** for enrollment as study participants: | Patients who meet ***any*** of these criteria are **not *eligible*** for enrollment as study participants: |
| - Female, age ≥45 years | - Bilateral breast reduction with nipple graft - Breast cancer or history of breast cancer - Pregnancy or lactation - Active breast infection - Lifetime risk ≥ 20% for breast cancer |

**Eligibility criteria for high-risk cohort**

| **Inclusion Criteria** | **Exclusion Criteria** |
| --- | --- |
| Patients must meet ***all*** of the following criteria ***to be eligible*** for enrollment as study participants: | Patients who meet ***any*** of these criteria are **not *eligible*** for enrollment as study participants: |
| - Female, age ≥18 years - Lifetime risk ≥ 20% for breast cancer (due to family history or high-risk breast cancer susceptibility genes) | - Bilateral breast reduction with nipple graft - Bilateral ablative breast surgery - Pregnancy or lactation (temporary exclusion criteria) - Active breast infection - Disseminated breast cancer |
