# Supplementary material for: Patient-centered research: how do women tolerate nipple fluid aspiration as a potential screening tool for breast cancer?
Source: BMC Cancer. 2022 Jun 27;22:705. doi: 10.1186/s12885-022-09795-8 (PMC9235076; doi:10.1186/s12885-022-09795-8)
Supplement: Supplementary file 4 — Additional file 4. Supplementary File 2. Discomfort questionnaire [file 12885_2022_9795_MOESM4_ESM.docx]

**Supplementary File 2- Discomfort questionnaire^[[1]](#endnote-2)^**

1. **Have you ever breastfed?**

- Yes
- No

1. **Can you indicate the degree of discomfort of breastfeeding on a scale of 0 (no discomfort at all) tot 10 (the worst I can imagine)?**

| 0 | 1 | 2 | 3 | 4 | 5 | 6 | 7 | 8 | 9 | 10 | N.A. |
| --- | --- | --- | --- | --- | --- | --- | --- | --- | --- | --- | --- |

1. **How long ago have you breastfed for the last time?**^1^ _________
2. **Have you ever had spontaneous nipple discharge (apart from periods of breastfeeding)?**

- Yes
- No

1. **Have you ever had a mammogram?**

- Yes
- No

1. **Can you indicate the degree of discomfort of the mammogram on a scale from 0 (no discomfort at all) to 10 (the worst you can imagine)?**

| 0 | 1 | 2 | 3 | 4 | 5 | 6 | 7 | 8 | 9 | 10 | N.A. |
| --- | --- | --- | --- | --- | --- | --- | --- | --- | --- | --- | --- |

1. **How long ago have you had a mammogram for the last time?**^1^ _________
2. **Have you ever had a breast MRI?**

- Yes
- No

1. **How long ago have you had a breast MRI for the last time?**^1^ _________
2. **Can you indicate the degree of discomfort of the MRI on a scale from 0 (no discomfort at all) to 10 (the worst you can imagine)?**

| 0 | 1 | 2 | 3 | 4 | 5 | 6 | 7 | 8 | 9 | 10 | N.A. |
| --- | --- | --- | --- | --- | --- | --- | --- | --- | --- | --- | --- |

1. **Have you ever had a breast physical exam in a hospital or at the general practitioner’s office?**

- Yes
- No

1. **Can you indicate the degree of discomfort of the physical examination on a scale from 0 (no discomfort at all) to 10 (the worst you can imagine)?**

| 0 | 1 | 2 | 3 | 4 | 5 | 6 | 7 | 8 | 9 | 10 | N.A. |
| --- | --- | --- | --- | --- | --- | --- | --- | --- | --- | --- | --- |

1. **How long ago have you had a breast physical exam for the last time?** ^1^_______
2. **If there was anything less pleasant or discomfortable during the study visit, what was it?**

**14a. If yes, Is there a way we might prevent this, or make it less discomfortable?**

1. **Would you undergo the procedure again:**
   1. In the context of study trial?^[[2]](#footnote-2)^
   2. If this would be a standard screening method?^1^
2. **Would you recommend the procedure to someone else, if this would be a standard screening method?**
3. In the context of study trial? ^1^
4. If this would be a standard screening method? ^1^

**Please circle in every row the number that best fits the discomfort you experienced during the procedure.**

|  |  | | | | | | | | | | |  |
| --- | --- | --- | --- | --- | --- | --- | --- | --- | --- | --- | --- | --- |
| **Prior to the procedure** |  | | | | | | | | | | |  |
| Waiting | 0 | 1 | 2 | 3 | 4 | 5 | 6 | 7 | 8 | 9 | 10 |  |
| Insecure about what will happen | 0 | 1 | 2 | 3 | 4 | 5 | 6 | 7 | 8 | 9 | 10 | N.A. |
| Questionnaire | 0 | 1 | 2 | 3 | 4 | 5 | 6 | 7 | 8 | 9 | 10 |  |
| **During the procedure** |  | | | | | | | | | | |  |
| Application of the anaesthetic cream, cleansing of the nipple | 0 | 1 | 2 | 3 | 4 | 5 | 6 | 7 | 8 | 9 | 10 | N.A. |
| Nasal spray | 0 | 1 | 2 | 3 | 4 | 5 | 6 | 7 | 8 | 9 | 10 | N.A. |
| Vacuum aspiration | 0 | 1 | 2 | 3 | 4 | 5 | 6 | 7 | 8 | 9 | 10 |  |
| Blood draw | 0 | 1 | 2 | 3 | 4 | 5 | 6 | 7 | 8 | 9 | 10 | N.A. |
| **Overall during the procedure** |  | | | | | | | | | | |  |
| Duration | 0 | 1 | 2 | 3 | 4 | 5 | 6 | 7 | 8 | 9 | 10 |  |
| Pain | 0 | 1 | 2 | 3 | 4 | 5 | 6 | 7 | 8 | 9 | 10 |  |
| Shame | 0 | 1 | 2 | 3 | 4 | 5 | 6 | 7 | 8 | 9 | 10 |  |
| **After the procedure** |  | | | | | | | | | | |  |
| Pain | 0 | 1 | 2 | 3 | 4 | 5 | 6 | 7 | 8 | 9 | 10 |  |
| **Overall discomfort of the study visit** | | | | | | | | | | | |  |
| Discomfort | 0 | 1 | 2 | 3 | 4 | 5 | 6 | 7 | 8 | 9 | 10 |  |

N.A.: not applicable

1. This is a selection of questions regarding discomfort included in the questionnaire. Other questions regarding anthropomorphic characteristics were removed for this supplementary file to keep the overview. [↑](#endnote-ref-2)
2. This question was added in the end of 2019. [↑](#footnote-ref-2)
